# Emerging risk factors for cognitive dysfunction among 1036 community‐dwelling older nigerian africans: Data from the valiant study

**DOI:** 10.1002/alz70860_104220

**Published:** 2025-12-23

**Authors:** Oladotun V Olalusi, Gabriel O Ogunde, Tolulope O Akinyemi, Joseph O Yaria, Eniola O Cadmus, Femi O Popoola, Mayowa Ogunronbi, Dorcas Olujobi, Olaoluwa Famuyiwa, Joshua O Akinyemi, Mayowa O Owolabi, Roman Romero‐Ortuno, Brian Lawlor, Raj Kalaria, Adesola Ogunniyi, Rufus O. Akinyemi

**Affiliations:** ^1^ Department of Neurology, University College Hospital, Ibadan, Oyo, Nigeria; ^2^ Neuroscience and ageing research unit, Institute of Advanced Medical Research and Training (IAMRAT), College of Medicine, University of Ibadan, Ibadan Nigeria, Oyo, Nigeria; ^3^ John P. Hussman Institute for Human Genomics, University of Miami, Miami, FL, USA; ^4^ Institute for Advanced Medical Research and Training, College of Medicine, University of Ibadan, Ibadan, Oyo, Nigeria; ^5^ Lead City University, Ibadan, Oyo, Nigeria; ^6^ University College Hospital, Ibadan, Oyo, Nigeria; ^7^ University of Ibadan and University College Hospital, Ibadan, Oyo, Nigeria; ^8^ University of Ibadan College of Medicine, Ibadan, Oyo, Nigeria; ^9^ College of Medicine, University of Ibadan, Ibadan, Oyo, Nigeria; ^10^ Institute of Advanced Medical Research and Training (IAMRAT), College of Medicine, University of Ibadan, Ibadan Nigeria, Oyo, Nigeria; ^11^ Neuroscience And Ageing Research Unit, Institute for Advanced Medical Research and Training, College of Medicine, University of Ibadan, Ibadan, Nigeria; ^12^ College of Medicine, University of Ibadan, Ibadan, Oyo State, Nigeria; ^13^ Global Brain Health Institute, Trinity College, Dublin, Ireland; ^14^ Global Brain Health Institute, Trinity College Dublin, Dublin, Ireland; ^15^ St James Hospital, Dublin, Ireland; ^16^ Newcastle University, Translational and Clinical Research Institutetitute for Ageing and Health, Newcastle UniversityNewcastle University, Newcastle upon Tyne, United Kingdom; ^17^ Africa Dementia Consortium, Ibadan, Ibadan, Nigeria; ^18^ Institute of Advanced Medical Research and Training, College of Medicine, University of Ibadan, Ibadan, Oyo State, Nigeria

## Abstract

**Background:**

Multi‐pronged risk factor characterization and primary prevention of dementia is cost‐effective. The Lancet 2024 commission on dementia observed that almost half of the burden of dementia is potentially preventable by tackling 14 putative risk factors. We examined the contribution of these risk factors to dementia among 1036 community‐dwelling older Nigerians.

**Method:**

The Vascular heAlth, fraiLty, and cognItion in Ageing Nigerians (VALIANT) is a longitudinal community‐based cohort study. One thousand and thirty‐six (1036) participants were recruited from Wards 2 and 3 of the Ibadan Northeast local government area of Oyo State, Nigeria through a multi‐stage, stratified cluster random sampling method. Patients were diagnosed as having dementia and MCI via a consensus diagnosis of at least two neurologists. Ten of the recently published 14 risk factors for dementia were identified. A multinomial logistic regression analysis was used to explore the relationship between emerging dementia risk factors and cognitive impairment. The aOR (95%CI) were reported.

**Result:**

The mean age was 65.2 (±10.5) with 27.2% males. A total of 35 participants (3.4%) and 112 (11%) had dementia and mild cognitive impairment respectively. The mean age (SD) of participants was 77.0 (11.3) dementia, 72.6 (9.8) MCI and 63.8 (9.9) normal. On the bivariate analysis, older age, female gender, low level of education and being underweight were associated with cognitive impairment. Of all vascular risk factors, only presence of HTN was significantly associated with dementia. The independent determinants of cognitive dysfunction aORs (95% CI) were older age 7.15 (3.73 – 13.69), female gender 1.70 (1.10 – 2.65), any form of education 0.18 (0.13 – 0.27), obesity/overweight 0.34 (0.18 – 0.64) and high social network score 0.95 (0.93 ‐0.97).

**Conclusion:**

In this cohort, low level of education, low BMI (indicative of poor physical health/frailty) and presence of social isolation showed an independent relationship with cognitive impairment. In Nigeria, besides documented traditional vascular risk factors, population‐wide strategies to curb the burden of dementia should focus on improved education, improved physical health and social integration. The understanding of the contribution of known risk factors to dementia burden should be context‐specific.

